# Scrutinizing transport phenomena and recombination mechanisms in thin film Sb_2_S_3_ solar cells

**DOI:** 10.1038/s41598-024-56041-1

**Published:** 2024-05-30

**Authors:** Z. Younsi, F. Meddour, H. Bencherif, M. Khalid Hossain, Latha Marasamy, P. Sasikumar, M. S. Revathy, Suresh Ghotekar, Mohammad R. Karim, Manikandan Ayyar, Rajesh Haldhar, Mirza H. K. Rubel

**Affiliations:** 1 LEREESI, Laboratory HNS-RE2SD, 05078 Batna, Algeria; 2 Institute of Electronics, Atomic Energy Research Establishment, Bangladesh Atomic Energy Commission, Dhaka, 1349 Bangladesh; 3 Department of Advanced Energy Engineering Science, Interdisciplinary Graduate School of Engineering Sciences, Kyushu University, Fukuoka, 816-8580 Japan; 4 Facultad de Química, Materiales-Energía, Universidad Autónoma de Querétaro, C.P.76010 Santiago de Querétaro, Querétaro, México; 5 Department of Physics, Saveetha School of Engineering, Saveetha Institute of Medical and Technical Sciences, Chennai, 602105 India; 6 Department of Physics, School of Advanced Sciences, Kalasalingam Academy of Research and Education, Krishnankoil, Tamil Nadu 626126 India; 7 Centre for Herbal Pharmacology and Environmental Sustainability, Chettinad Hospital and Research Institute, Chettinad Academy of Research and Education, Kelambakkam, 603103 Tamil Nadu India; 8 Department of Mechanical Engineering, College of Engineering, King Saud University, 11421 Riyadh, Saudi Arabia; 9 Department of Chemistry, Centre for Material Chemistry, Karpagam Academy of Higher Education, Coimbatore, Tamil Nadu 641 021 India; 10 School of Chemical Engineering, Yeungnam University, Gyeongsan, 38541 Republic of Korea; 11 Department of Materials Science and Engineering, University of Rajshahi, Rajshahi, 6205 Bangladesh

**Keywords:** Sb_2_S_3_ solar cells, Recombination mechanisms, Analytical modeling, Device optimization, Optics and photonics, Physics

## Abstract

The Schockley–Quisser (SQ) limit of 28.64% is distant from the Sb_2_S_3_ solar cells’ record power conversion efficiency (*PCE*), which is 8.00%. Such poor efficiency is mostly owing to substantial interface-induced recombination losses caused by defects at the interfaces and misaligned energy levels. The endeavor of this study is to investigate an efficient Sb_2_S_3_ solar cell structure via accurate analytical modeling. The proposed model considers different recombination mechanisms such as non-radiative recombination, Sb_2_S_3_/CdS interface recombination, Auger, SRH, tunneling-enhanced recombination, and their combined impact on solar cell performance. This model is verified against experimental work (Glass/ITO/CdS/Sb_2_S_3_/Au) where a good coincidence is achieved. Several parameters effects such as thickness, doping, electronic affinity, and bandgap are scrutinized. The effect of both bulk traps located in CdS and Sb_2_S_3_ on the electrical outputs of the solar cell is analyzed thoroughly. Besides, a deep insight into the effect of interfacial traps on solar cell figures of merits is gained through shedding light into their relation with carriers’ minority lifetime, diffusion length, and surface recombination velocity. Our research findings illuminate that the primary contributors to Sb_2_S_3_ degradation are interfacial traps and series resistance. Furthermore, achieving optimal band alignment by fine-tuning the electron affinity of CdS to create a Spike-like conformation is crucial for enhancing the immunity of the device versus the interfacial traps. In our study, the optimized solar cell configuration (Glass/ITO/CdS/Sb_2_S_3_/Au) demonstrates remarkable performance, including a high short-circuit current (*J*_*SC*_) of 47.9 mA/cm^2^, an open-circuit voltage (*V*_*OC*_) of 1.16 V, a fill factor (*FF*) of 54%, and a notable improvement in conversion efficiency by approximately 30% compared to conventional solar cells. Beyond its superior performance, the optimized Sb_2_S_3_ solar cell also exhibits enhanced reliability in mitigating interfacial traps at the CdS/Sb_2_S_3_ junction. This improved reliability can be attributed to our precise control of band alignment and the fine-tuning of influencing parameters.

## Introduction

Nowadays, renewable energy sources contribute to approximately 17% of the world’s total energy consumption^1^. Among these sources, solar energy ranks as the third most prominent, trailing only hydropower and wind energy^2–6^. Photovoltaic (PV) solar cells are a reliable way to convert solar energy into electricity. These devices are essential in lowering the carbon footprint of our energy infrastructure in addition to producing pure, eco-friendly energy^7^.

Currently, crystalline silicon (c-Si) holds a commanding 95% market share worldwide in the photovoltaic (PV) industry^8,9^. Nevertheless, there are several inherent limitations in Si-PV technology, such as its low absorption coefficient (α = 103 cm^−1^), stiffness, bulkiness, costly production, and indirect and narrow-bandgap properties (Eg = 1.1 eV)^10^. As Si solar cells approach their theoretical efficiency limitation of 29.4%, the photovoltaic society is being forced to seriously look into substitute photovoltaic technologies. These substitutes are intended to supplement or substitute Si-solar cells, which are more expensive. Although they have been produced and brought to market, solar energy systems based on Cu(In, Ga)Se_2_ (CIGS) and CdTe have a lesser share of the market of about 5%^11^.

However, the toxic effects of cadmium (Cd) and the scarcity of tellurium (Te), indium (In), and gallium (Ga) make them less viable for broad multi-terawatt installation^12^. Because of their high absorption coefficient (α ∼ 105 cm^−1^ in the visible region), reliable physicochemical characteristics, solution processing ability, and use of earthly-abundant and non-toxic materials, Sb_2_S_3_ solar cells have garnered significant focus within the PV society^13,14^. With a distinct quasi-one-dimensional (Q1D) crystal structure that provides remarkable flexibility, self-passivated grain boundaries, and defect tolerance, Sb_2_S_3_ crystallizes in a single, stable orthorhombic phase^15,16^. It is suited for indoor photovoltaics (IPV) uses, especially for Internet of Things (IoT) sensors, because of its remarkable efficiency in low and ambient light. Furthermore, Sb_2_S_3_ is a promising replacement for amorphous Silicon (a-Si) in low-power devices such as wristwatches and pocket calculators^17,18^.

Despite extensive research efforts in the Sb_2_(S, Se)_3_ solar cell field^19^, reported efficiencies remain relatively low, which is a common challenge faced by many emerging solar cell technologies. In particular, efficiency values for Sb_2_S_3_-based inorganic solar cells have been found to be less than 7%^20^, which is notably less than what has been recorded for CdTe^21^, CIGS, and perovskite solar cells^22–25^. It has been established that for thin-film solar cells, mechanisms like I-V hysteresis, buffer/absorber interface recombination, tunneling-enhanced recombination, and recombination due to bulk defects may have primary impacts on device efficiency^26^. The reasons behind these low efficiencies are still unknown.

On the other hand, little is known about how these transport pathways affect Sb_2_S_3_ solar cells, which calls for more research. Sb_2_S_3_ solar cells can obtain efficiency values of roughly 26% under the radiative limit, however, defects in the absorber material decrease efficiency to less than 11%, according to a new early study to comprehend Sb_2_S_3_-based solar cells^27^. It has been demonstrated that the mechanisms for transport are essential in other technologies, such as Kesterite solar cells. As a result, this paper presents an in-depth theoretical examination of Sb_2_S_3_ solar cells, including an assessment of the various transport mechanisms—such as tunneling-enhanced recombination, Sb_2_S_3_/CdS interface recombination, and non-radiative recombination—and their combined effects on solar cell parameters. Additionally, the influence of shunt and series resistances, as well as various minority carrier lifetime values and Sb_2_S_3_/CdS interface recombination speeds, are investigated.

It is important to note that instead of concentrating on the main constraints of the technology, numerous studies on solar cell modeling for CdTe, CIGS, and Kesterite structures frequently highlight calibration specifics. Hence, the primary novelty of this work lies in its detailed examination of the limitations of Sb_2_S_3_ solar cell technology. The findings show that our proposed model can explain the relatively low-efficiency values stated for Sb_2_S_3_ solar cells when compared with experimental data from the literature^28^. It is discovered that a number of variables mainly limit the efficiency of Sb_2_S_3_ solar cells. Initially, to surpass the 8% efficiency barrier, efforts must be directed toward minimizing the impact of series and shunt resistances. Subsequently, a second efficiency barrier at around 10% is identified, where defects in the Sb_2_S_3_ bulk and Sb_2_S_3_/CdS interface become dominant factors affecting performance. The enhanced structure achieves a V_OC_ of 1.16 V via band alignment tuning by establishing a Spike-like conformation and global design optimization, outperforming the baseline in terms of open circuit voltage.

Finally, this study outlines the objectives of conducting a thorough analysis of Sb_2_S_3_ solar cells, with a specific focus on transport mechanisms and their influence on solar cell parameters. By systematically addressing efficiency barriers, including resistances and defects in the Sb_2_S_3_ structure, the research aims to achieve its objectives of advancing the performance and overall efficiency of Sb_2_S_3_ solar cells. Ultimately, the study seeks to pave the way for the development of more efficient Sb_2_S_3_ solar cell technology, contributing valuable insights to the field of photovoltaics.

The novelty of this work lies in its detailed theoretical examination of Sb_2_S_3_ solar cells, specifically focusing on the intricate interplay of various transport mechanisms such as tunneling-enhanced recombination, Sb_2_S_3_/CdS interface recombination, and non-radiative recombination. By thoroughly assessing these mechanisms and their combined effects on solar cell parameters, the work offers new insights into the underlying processes governing the performance of Sb_2_S_3_-based photovoltaic devices. This contributes to advancing the understanding of the fundamental physics and engineering principles involved in such solar cells, potentially paving the way for enhanced design strategies and improved efficiency in future photovoltaic technologies.

Different sections of this paper are arranged as follows: a thorough description of the recommended Sb_2_S_3_ solar cell and the necessary procedures for building the associated modeling framework are given in “Transport mechanisms and device structure”. We discuss and show the outcomes of our modeling in “Results and discussion”. Our results and recommendations for further research directions are presented in “Conclusions”.

## Transport mechanisms and device structure

Chalcogenide solar cells employ a complex interplay of transport mechanisms to efficiently convert sunlight into electricity. Drift and diffusion processes guide the movement of charge carriers, while recombination and generation play pivotal roles in creating and reducing electron–hole pairs. Surface recombination at material interfaces and the presence of traps or defects within the semiconductor can significantly impact the device’s performance. Tunneling may occur at heterojunctions, affecting charge carrier collection and extraction. The choice of Ohmic or Schottky contacts on the solar cell’s surface also influences charge carrier collection efficiency. Understanding and optimizing these mechanisms is crucial for enhancing the efficiency, stability, and overall performance of chalcogenide solar cells.

**Figure 1:**
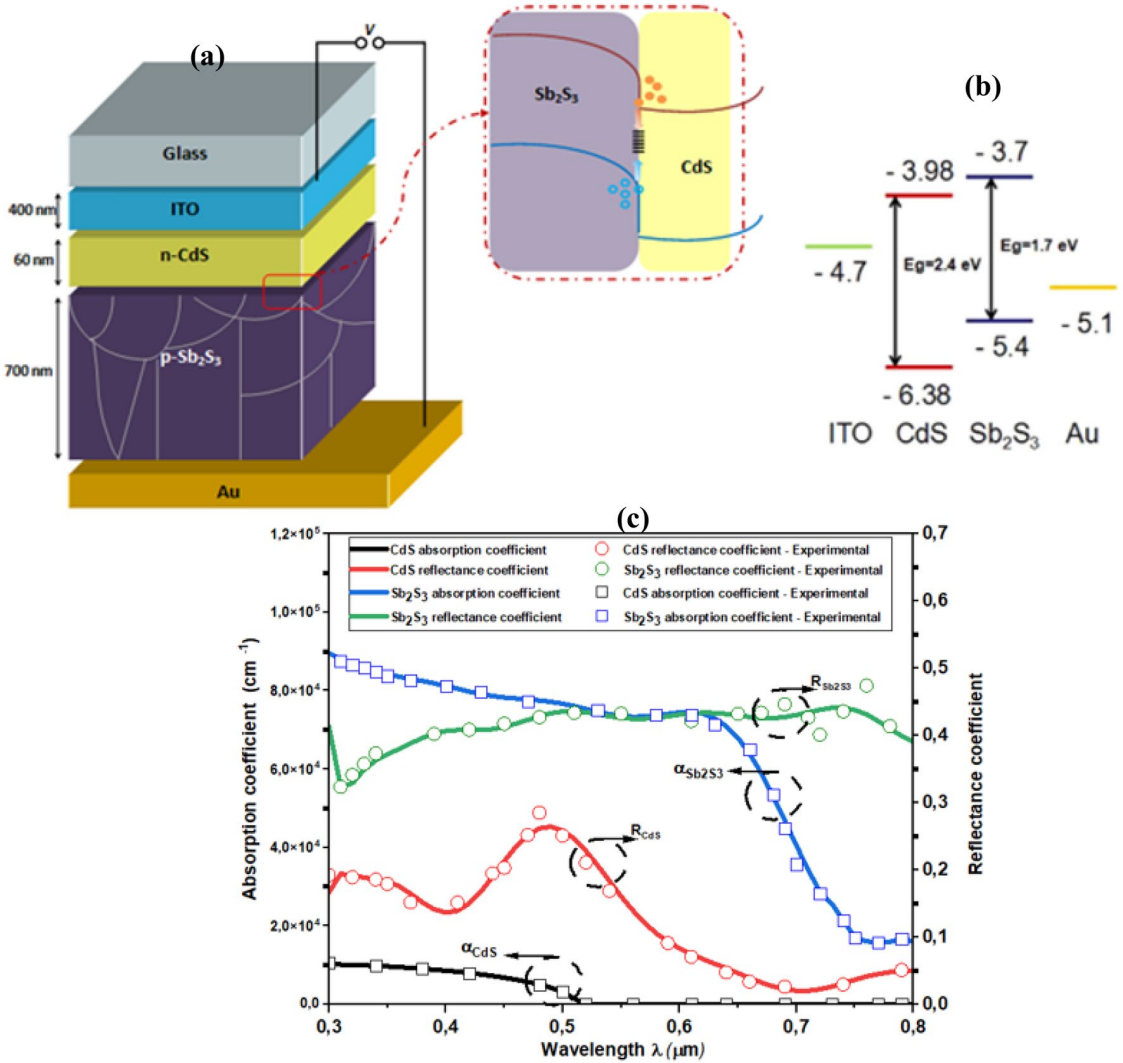
(**a**) Conventional structure of the device. (**b**) Device band diagram energy at equilibrium. Reproduce with permission from Ref.^28^. (**c**) Absorption coefficients of different buffer materials. Reproduce with permission from Ref.^29^.

The aim of this study is to develop an accurate model that can give a deeper understanding of these transport mechanisms and their intricate interactions, insights about the dominant recombination mechanism, and an efficient strategy to optimize the device in terms of efficiency. Figure 1 shows the conventional device structure; device band diagram energy at equilibrium, and absorption coefficients of different buffer materials. The input parameters used to model the conventional Sb_2_S_3_ SC are summarized in Table 1. The bulk defect properties utilized in the modeling framework are listed in Table 2.

**Table 1:** Input data^28,30–32^.

Parameters	Unit	CdS		Sb_2_S_3_	
Type of layers		n-type	Ref	p-type	Ref
Thickness	nm	60	^28^	700	^28^
Dielectric constant (ε_r_)	–	9	^30^	5 or 7.08	^31,32^
Electron affinity	eV	3.98	^28^	3.7	^28,32^
Band-gap	eV	2.4	^28,31^	1.7	^28,32^
E_C_	eV	− 3.98	^28^	− 3.7	^28^
E_V_	eV	− 6.38	^28^	− 5.4	^28^
Thermal velocity of holes	cm s^−1^	10^7^	^31^	10^7^	^32^
Thermal velocity of electrons	cm s^−1^	10^7^		10^7^	^32^
Electron mobility (µ_n_)	cm^2^/V s			0.8	^32,33^
Hole mobility (µ_p_)	cm^2^/V s	50	^31^	0.2	^32,33^
Electron effective mass, (m_e_*/m_0_)	–	0.25	^31^	1.035	^31^
Hole effective mass, (m_h_*/m_0_)	–	0.7	^31^	1.843	^31^
Effective density of states (DOS) in CB (N_C_)	cm^−3^	1.8 × 10^19^	^31^	5 × 10^19^	^32^
Effective density of states (DOS) in VB (N_V_)	cm^−3^	2.4 × 10^18^	^31^	10^20^	^32^
Acceptor concentration (N_A_)	cm^−3^	0		4.7 × 10^15^	^30^
Donor concentration (N_D_)	cm^−3^	10^17^	^31^	0

**Table 2:** Bulk defect parameters.

Parameters/layer types	CdS^31^	Sb_2_S_3_^32^
Capture cross section holes (cm^2^)	10^−13^	10^−13^
Capture cross section electrons (cm^2^)	10^−17^	10^−14^
Total defect density (cm^−3^)	10^17^	10^13^–10^19^

### Optical properties

Antimony sulfide (Sb_2_S_3_) solar cells exhibit promising optical properties for photovoltaic applications. Sb_2_S_3_ is known for its strong light absorption in the visible and near-infrared spectrum, allowing efficient utilization of sunlight. This property, combined with its earth-abundant and non-toxic nature^34^, positions Sb_2_S_3_ as a compelling material for the development of sustainable and cost-effective solar energy technologies.

The incident power (*P*_*i*_) is calculated as the integral of the solar spectral irradiance on the ground (*IRS (λ)*)^35^:

1 $${P}_{i}={\int }_{{\lambda }_{min}}^{{\lambda }_{max}}IRS\left(\lambda \right)d\lambda$$

The analytical expression of *IRS*(*λ*) takes the form of^35^:

2 $$IRS\left(\lambda \right)=0.06977+{7.0625\left[1-exp(\frac{-(\lambda -0.26053)}{0.15994})\right]}^{2.28411}exp(\frac{-(\lambda -0.26053)}{0.22285})$$

which denotes the finest fit of the real solar spectral irradiance behavior in the VS and IR wavelength range as described in^35^.

The absorption coefficient $\alpha$ for both materials CdS and Sb_2_S_3_ are in the form of:

3 $${\alpha }_{CdS}={\alpha }_{1}=\frac{A}{E}{(E-{E}_{g\_CdS})}^{1/2}$$

4 $${\alpha }_{Sb2S3}={\alpha }_{2}=\frac{B}{E}{(E-{E}_{g\_Sb2S3})}^{1/2}$$

where *A* and *B* are constants, $E$ is the photon energy, ${E}_{g\_CdS}$ and ${E}_{g\_Sb2S3}$ are the band gaps of CdS and Sb_2_S_3_, respectively.

The absorption and the reflectance coefficient of CdS and Sb_2_S_3_ are estimated from experimental results^29,36^.

### Photocurrent density

In the thin film solar cell under consideration, we can calculate the solar cell photocurrent density as shown by solving Poisson’s equation and the carrier continuity equations for each device region, involving the first neutral region, the space charge region, and the second neutral region^37^. The total photocurrent density is given as follows:

5 $${J}_{ph,T}\left(\lambda ,R\right)={J}_{ph,E}\left(\lambda ,R\right)+{J}_{ph,D}\left(\lambda ,R\right)+{J}_{ph,B}(\lambda ,R)$$

where ${J}_{ph,E}$, ${J}_{ph,D}$, and ${J}_{ph,B}$ represent the light-generated current density in the emitter layer, junction space-charge region, and base region, respectively. After performing certain mathematical transformations, the photocurrent density for each region can be described as follows:

For the n-type CdS region (emitter layer)^37^.

6 $${J}_{ph,E}\left(\lambda ,R\right)=\left[\left(q\frac{F(1-R){\alpha }_{CdS}{L}_{p}}{{\alpha }_{CdS}^{2}{L}_{p}^{2}-1}\right)\times \left(\frac{\left(\frac{{S}_{p}{L}_{p}}{{D}_{p}}+{\alpha }_{CdS}{L}_{p}\right)-exp\left(-{\alpha }_{CdS}{x}_{j}\right)\left(\frac{{S}_{p}{L}_{p}}{{D}_{p}}cosh\left(\frac{{x}_{j}}{{L}_{p}}\right)+sinh\left(\frac{{x}_{j}}{{L}_{p}}\right)\right)}{\frac{{S}_{p}{L}_{p}}{{D}_{p}}sinh\left(\frac{{x}_{j}}{{L}_{p}}\right)+cosh\left(\frac{{x}_{j}}{{L}_{p}}\right)}-{\alpha }_{CdS}{L}_{p}exp(-{\alpha }_{CdS}{x}_{j})\right)\right]$$

where *q* is the electric charge, *F* represents the flux of light, *x*_*j*_ is the emitter thickness, *D*_*p*_ and *L*_*p*_ refer to the carrier (minority holes) diffusion constant given by *D*_*p*_ = *V*_*t*_ × *µ*_*p*_, and diffusion length in the n-region given by *L*_*p*_ = *(D*_*p*_ × *τ*_*p*_*)*^*1/2*^, respectively. S_p_ is the effective recombination velocity on this layer (on the rear side).

For the space-charge region^37^.

7 $${J}_{ph,D}\left(\lambda ,R\right)=qF\left(1-R\right)exp\left(-{\alpha }_{CdS}{x}_{j}\right)\left[\left(1-exp\left(-{\alpha }_{CdS}{W}_{1}\right)\right)+exp\left(-{\alpha }_{CdS}{W}_{1}\right)\left(1-exp\left(-{\alpha }_{Sb2S3}{W}_{2}\right)\right)\right]$$

where *W*_*1*_ and *W*_*2*_ are the space-region edges.

For the p-type Sb_2_S_3_ region^37^.

8 $${J}_{ph,B}\left(\lambda ,R\right)=\left[\left(q\frac{F(1-R){\alpha }_{Sb2S3}{L}_{n}}{{\alpha }_{Sb2S3}^{2}{L}_{n}^{2}-1}\right)exp(-{\alpha }_{CdS}\left({x}_{j}+{w}_{1}\right)-{\alpha }_{Sb2S3}{w}_{2})\left({\alpha }_{Sb2S3}{L}_{n}-\frac{\frac{{S}_{n}{L}_{n}}{{D}_{n}}+\left(cosh\left(\frac{{H}^{\prime}}{{L}_{n}}\right)-exp\left(-{\alpha }_{Sb2S3}{H}^{\prime}\right)\right)+sinh\left(\frac{{H}^{\prime}}{{L}_{n}}\right)+{\alpha }_{Sb2S3}{L}_{n}exp\left(-{\alpha }_{Sb2S3}{H}^{\prime}\right)}{\frac{{S}_{n}{L}_{n}}{{D}_{n}}sinh\left(\frac{{H}^{\prime}}{{L}_{n}}\right)+cosh\left(\frac{{H}^{\prime}}{{L}_{n}}\right)}\right)\right]$$

where Hʹ is the emitter thickness, D_n_ and L_n_ refer to the carrier (minority electrons) diffusion constant given by *D*_*n*_ = *V*_*t*_ × *µ*_*n*_, and diffusion length in the p-region given by *L*_*n*_ = *(D*_*n*_ × *τ*_*n*_*)*^*1/2*^, respectively. *S*_*n*_ is the effective recombination velocity on this layer.

### Recombination mechanisms

Understanding recombination mechanisms is crucial for optimizing the efficiency of Sb_2_S_3_ solar cells. In this study, we aim to scrutinize fundamental processes such as Shockley–Read–Hall, Auger, and surface recombination, each intricately governing the dynamics of charge carriers within the Sb_2_S_3_ solar cell. Our investigation goes beyond the broad spectrum and delves into specific mechanisms, including tunneling-enhanced recombination and Sb_2_S_3_/CdS interface recombination. Tunneling-enhanced recombination involves the quantum phenomenon of carriers transporting through potential barriers, influencing recombination dynamics. Sb_2_S_3_/CdS interface recombination, on the other hand, focuses on the boundary between the Sb_2_S_3_ absorber and the CdS buffer layer, examining how charge carriers interact at this crucial interface. Simultaneously, we meticulously examine non-radiative recombination processes, where carriers recombine without emitting photons, leading to energy loss as heat. Non-radiative recombination processes can significantly impact carrier loss and overall solar cell efficiency. The primary thrust of this study is a meticulous determination of the dominant recombination mechanism. By navigating the complexities of these mechanisms, we aim to not only deepen the theoretical foundations but also provide actionable strategies for mitigating their impact. This dual-pronged approach seeks to elevate the efficiency and stability of Sb_2_S_3_ solar cells, positioning them at the forefront of sustainable and efficient photovoltaic technologies. The proposed experimental techniques aim to address and reduce the impact of these recombination mechanisms, ensuring practical viability and heightened performance in real-world applications.

#### Bulk recombination

The estimation of the effective minority carrier lifetime, denoted as *τ*_*n(p)*_, can be articulated as follows:^38^.

9 $$\frac{1}{{\tau }_{n(p)}}=\frac{1}{{\tau }_{n(p)B}}+\frac{1}{{\tau }_{s}}+\frac{1}{{\tau }_{Aug}}$$

where *τ*_*n(p)B*_*, τ*_*s,*_* τ*_*Aug*_, are the bulk recombination lifetime defect density, the surface recombination lifetime, and the Auger recombination lifetime, respectively. The bulk recombination lifetime defect density is given as *τ*_*n(p)B*_ = *(V*_*th*_ × *σ*_*n(p)*_ × *N*_*tn(p)*_*)*^−*1*^, where *σ*_*n(p)*_ refers to the capture cross-sections of traps, *N*_*tn(p)*_ refers to defect density.

#### Interface recombination

The lifetime at the CdS/Sb_2_S_3_ interface, influenced by interface recombination, is calculated as^39^ *τ*_*s*_ = *d/(2* × *S)*, where *d* represents the crystalline thickness. For the CdS/Sb_2_S_3_ interface, we can describe the surface recombination velocity *S* as *S* = *(V*_*th*_ × *σ*_*n(p)*_ × *D*_*i*_*)*, with σ_n(p)_ and *D*_*i*_ denoting the capture cross-sections of traps and interface state density at the interface, respectively.

#### Tunneling enhanced recombination

Tunneling recombination plays a vital role in the performance of solar cells when doping with n-type (electron-rich) and p-type (hole-rich) materials. In this context, doping introduces impurities into the semiconductor material, creating a junction that facilitates the separation of photogenerated electron–hole pairs. However, excessive doping can lead to tunneling recombination, where electrons and holes recombine prematurely through quantum mechanical tunneling, reducing the overall efficiency of the solar cell. Careful control of doping levels and the design of the junction are essential to strike the right balance between charge carrier separation and minimizing tunneling recombination, ultimately optimizing the solar cell’s energy conversion efficiency.

In our modeling framework, we incorporate the recombination rate expression developed by Hurkx et al.^40^ for tunneling-assisted recombination, which is articulated as follows:

10 $$R=\frac{np-{n}_{i}^{2}}{{\gamma }_{p}\left(n+{n}^{*}\right)+{\gamma }_{n}\left(p+{p}^{*}\right)}$$

In this context, *n* and *p* represent the quantities of free electrons and holes, respectively, *kT* denotes the thermal energy, *n*_*i*_ stands for the intrinsic carrier concentration, and n* and p* are defined as *N*_*C*_ × *exp *[*(E*_*T*_* − E*_*C*_*)/kT*] and *N*_*V*_ × *exp *[*(E*_*V*_* − E*_*T*_*)/kT*] respectively. Here, *N*_*C*_ and *N*_*V*_ are the effective density of states in the conduction and valence bands, and *E*_*T*_ represents the trap energy.

The term ${\gamma }_{n}$_*(p)*_ is defined as ${\gamma }_{n}$_*(p)*_ = *τ*_*n(p)*_*[1* + *Γ]*^−*1*^ where *τ*_*n(p)*_ signifies the lifetimes of trapped electrons (or holes), and *Γ* represents a correction factor accounting for the enhancement of recombination due to tunneling.

### Solar cell characteristics

*J*_*ph,T*_ is determined by integrating *J*_*ph,T*_*(λ, R)* over the entire solar spectrum range. When illuminated, the current–voltage *J (V)* characteristics of a solar cell can be described using the following equation^37^:

11 $$J\left(V\right)={J}_{s}\left[exp\,exp\left(\frac{V-J{R}_{s}}{{AV}_{t}}\right)-1\right]+\frac{V-J{R}_{s}}{{R}_{sh}}-{J}_{ph,T}$$

In this context, *J*_*s*_ represents the reverse dark current density at the depletion zone, which relies on the prevailing transport mechanisms. *J*_*ph,T*_ signifies the photocurrent density, while *R*_*s*_ and *R*_*sh*_ denote the series and shunt resistances, respectively. Additionally, *q* represents the charge of an electron, *A* stands for the ideality factor, *V* corresponds to the applied voltage, and Vt is the thermal voltage.

From these *J–V* characteristics, we can derive the short-circuit current density (*J*_*sc*_) and, by solving for *f* (*V*_*oc*_) = 0, determine the open-circuit voltage (Voc).

12 $${J}_{SC}\left(V=0\right)= {J}_{ph,T}$$

13 $${f(V}_{oc})={J}_{s}\left[exp\,exp\left(\frac{{V}_{oc}}{{AV}_{t}}\right)-1\right]+\frac{{V}_{oc}}{{R}_{sh}}-{J}_{ph,T}$$

Fill factor *FF* and the conversion efficiency are deduced by the following equations:

14 $$FF=\left[\frac{{P}_{max}}{{V}_{OC}{J}_{SC}}\right]$$

15 $$\eta =\left[\frac{{P}_{max}}{{P}_{i}}\right])={J}_{s}\left[exp\,exp\left(\frac{{V}_{oc}}{{AV}_{t}}\right)-1\right]+\frac{{V}_{oc}}{{R}_{sh}}-{J}_{ph,T}$$

## Results and discussion

### Model validation

Validation of the proposed Sb_2_S_3_ solar cell model against experimental data is a crucial step in affirming its accuracy and dependability^28^. This validation process serves to pinpoint any disparities or constraints in the model, ensuring its faithful representation of real-world performance. Ultimately, this validation serves as a vital link between theory and practical application, driving advancements in Sb_2_S_3_ solar cell technology.

**Figure 2:**
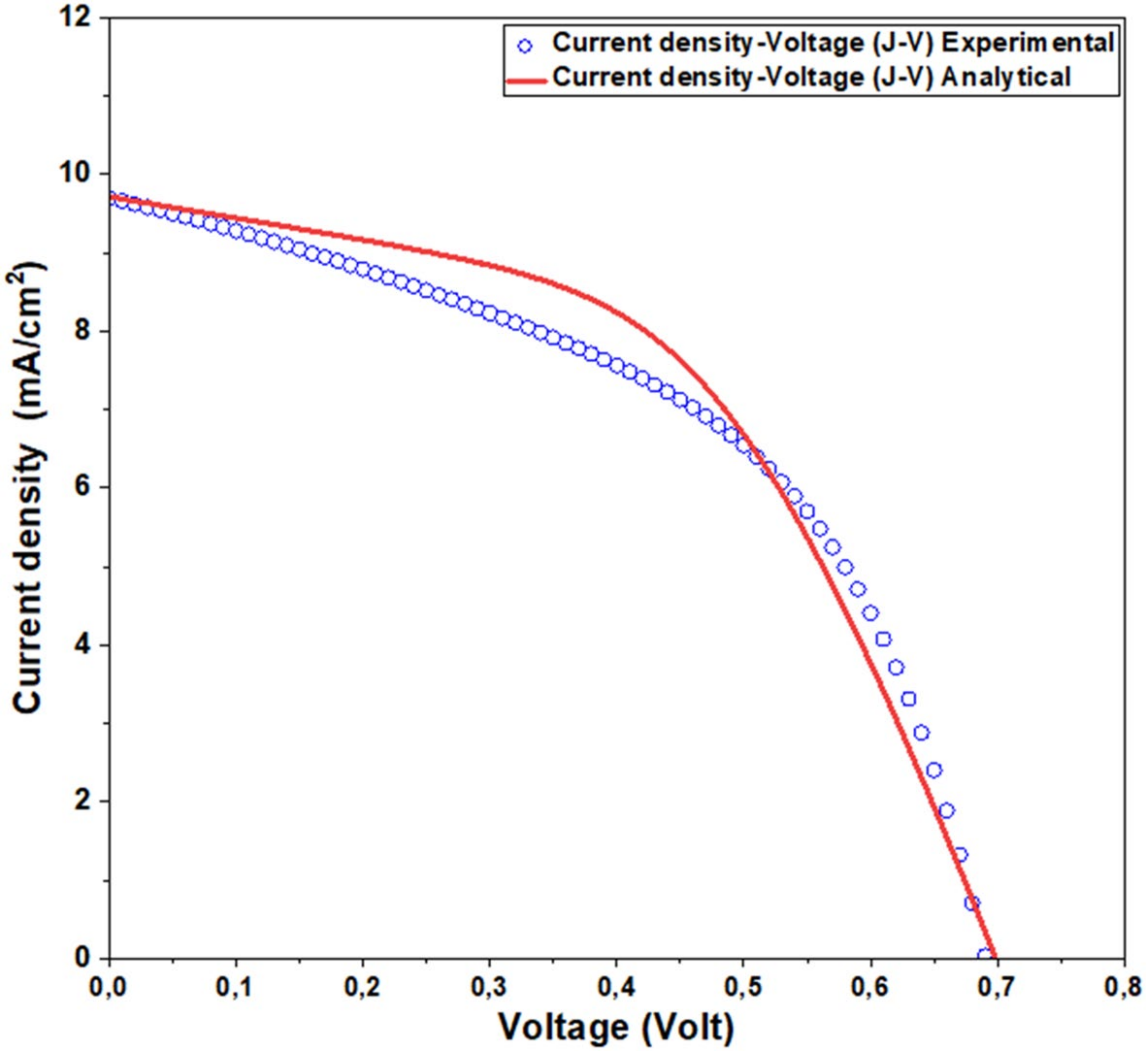
Comparison of the *I–V* characteristics between the experimental data and the proposed model of CdS/Sb_2_S_3_ solar cells.

To assess the precision of the framework, the J–V characteristic of the conventional Sb_2_S_3_ solar cell is checked against available experimental data^28^. A close fit is shown in Fig. 2, suggesting the accuracy of the proposed model. The main reasons for the slight variation that is observed are the accumulated charges at the contact and the series resistance. The primary figures of merit for the simulation results and the experimental data are contrasted in Table 3.

**Table 3:** Key performance metrics comparison of simulated and experimental Sb_2_S_3_ solar cells^28^.

	J_sc_ (mA/cm^2^)	V_oc_ (Volt)	FF (%)	R_sh_ (Ω)	R_s_ (Ω)	A
Exp. results^28^	10.8	0.71	47.5	351.5	18.4	1.9
Analytical results	9.72	0.70	50.63	351.5	18.4	1.9

### Effect of doping, thickness, bandgap, and affinity of CdS layer

**Figure 3:**
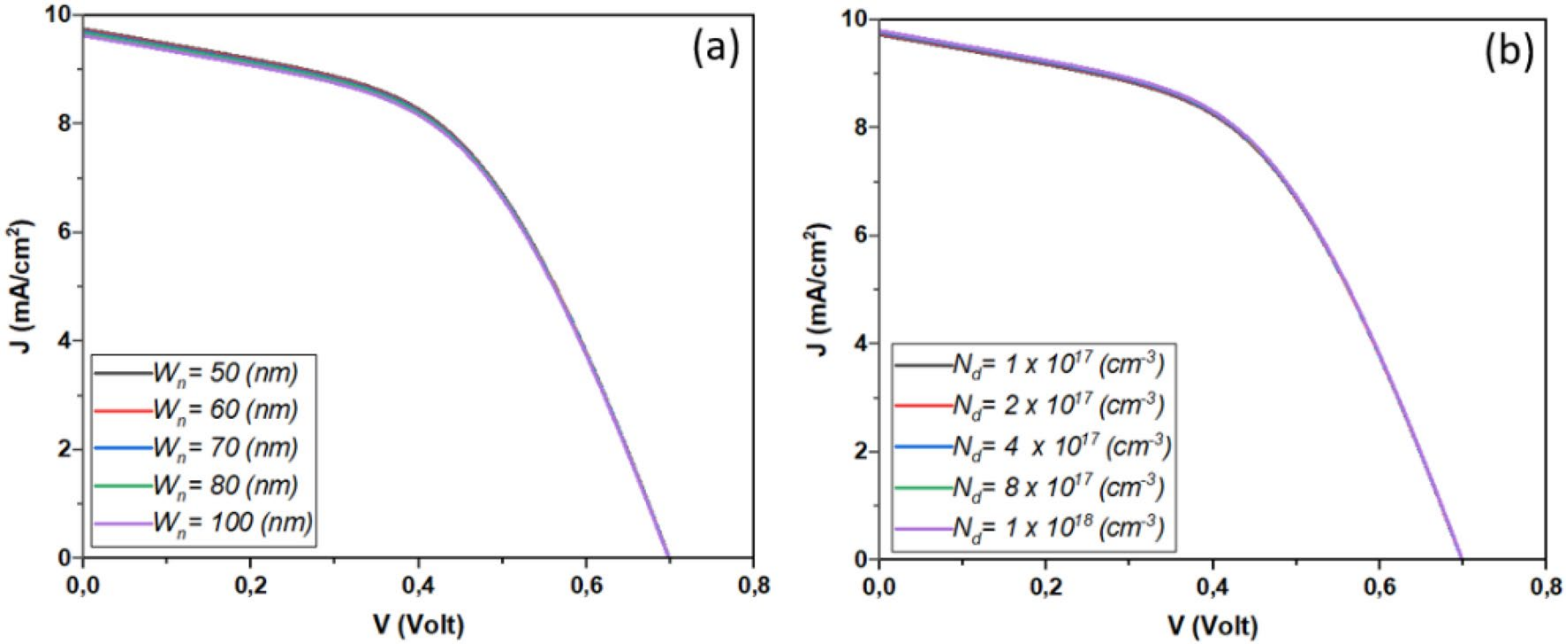
Effect of doping, thickness, bandgap, and affinity of CdS layer (**a**) *N*_*d*_ = 1. × 10^17^&*Wn* variable, (**b**) *N*_*d*_ variable & *Wn* = 60 nm.

**Figure 4:**
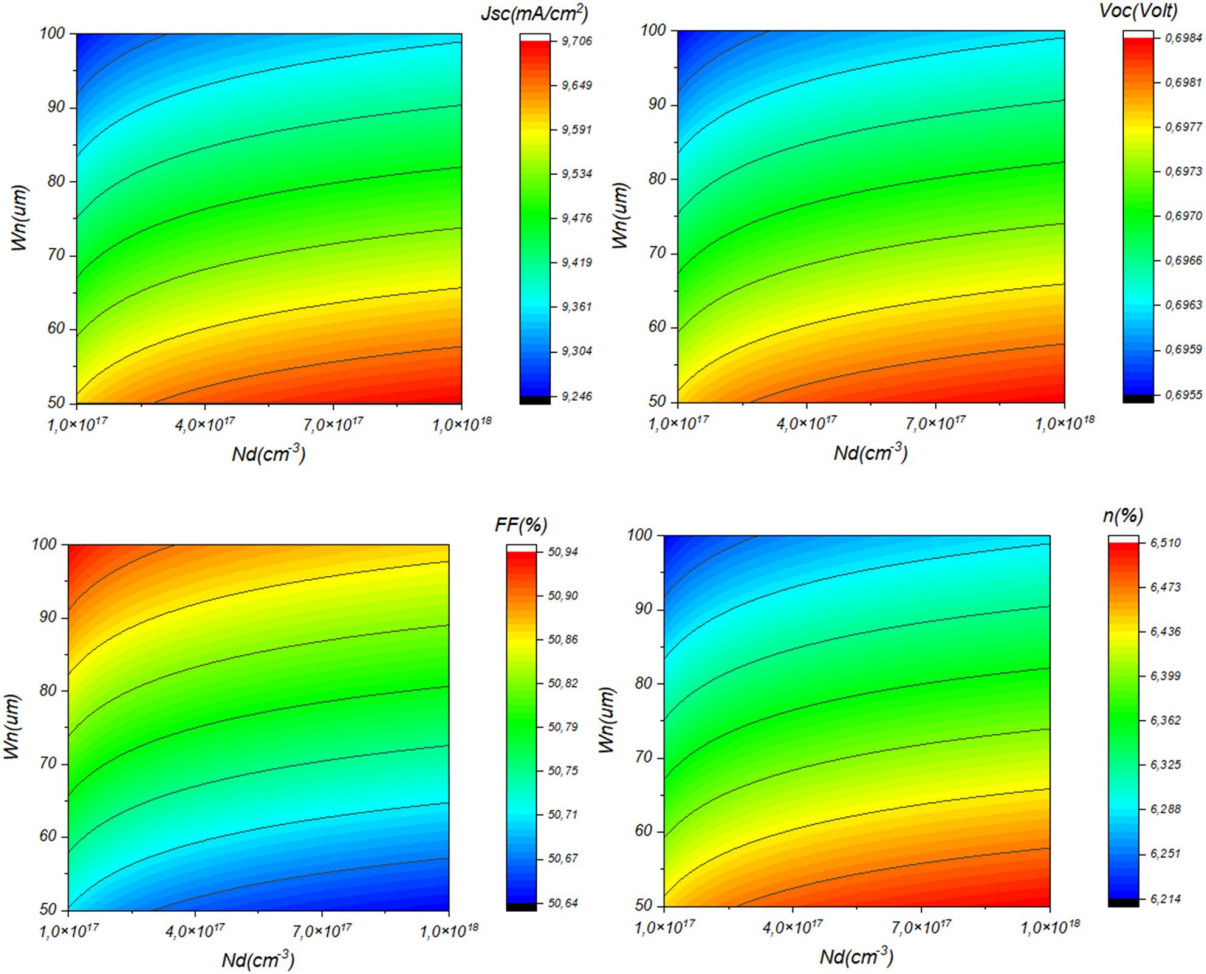
Effect of doping concentration and thickness of CdS layer on the performance of Sb_2_S_3_ solar cell.

The performance of Sb_2_S_3_ solar cells is influenced by factors such as CdS layer doping, thickness, bandgap, and affinity. These variables significantly impact efficiency and stability. Research into their effects is essential for improving solar cell design and efficiency and advancing sustainable photovoltaic technology. Figure 3 depicts the influence of CdS layer doping concentration and thickness on the performance of the investigated solar cell. This result is shown in detail in Fig. 4.

From Fig. 4, it is noticeable that high CdS layer doping concentration improves the device *J*_*SC*_ and *V*_*OC*_ due to the improved separation mechanism and fast collection^41–44^. High concentration causes an excursion of the depletion zone in the absorber layer which enhances the electric field. Besides, High doping concentration improves the layer conductivity and allows a swift move of the carriers to the electrode. Another insight from this figure is the effect of the CdS thickness, where the more the thickness increases the more the device performance will be hindered. This fact is due mainly to the penetration of light and the insufficient diffusion length caused carrier recombination phenomenon.

**Figure 5:**
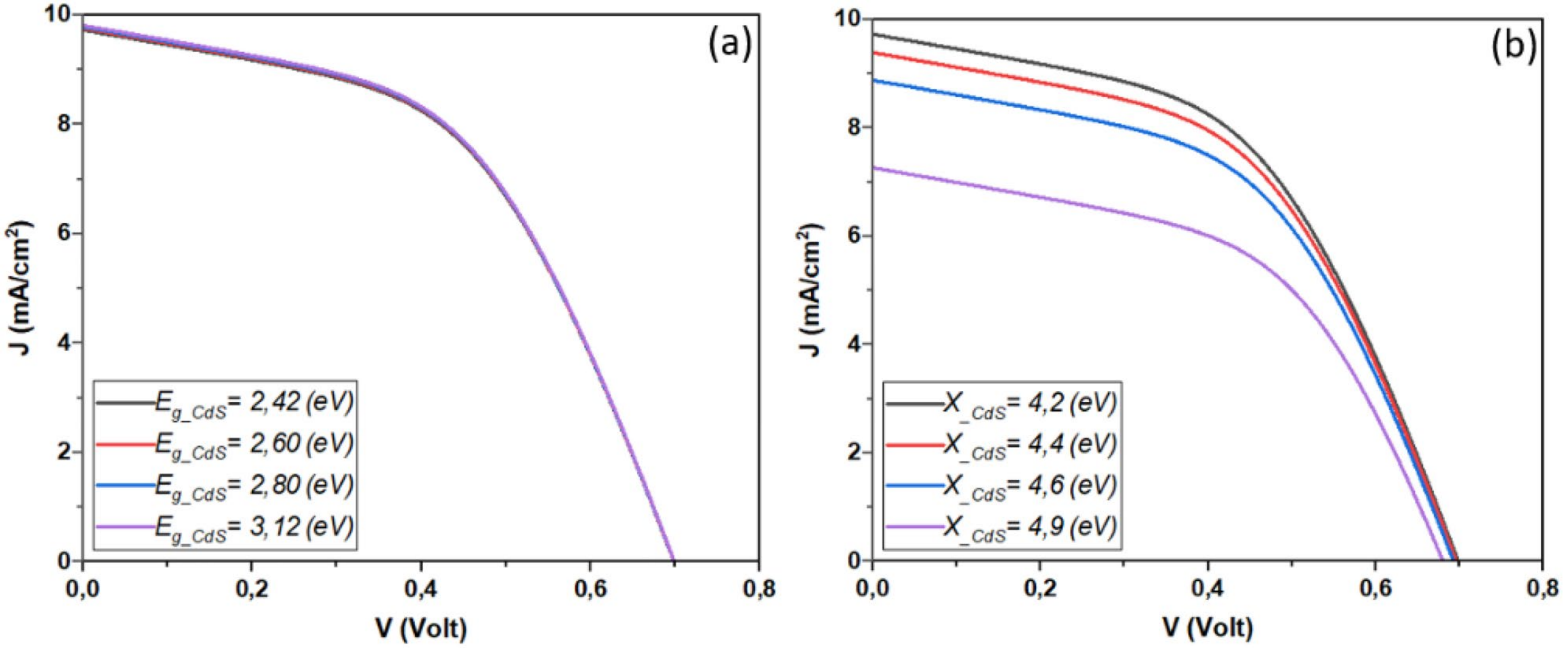
Effect of Doping, thickness, bulk traps and bandgap of Sb2S3, (**a**) Affinity of CdS = 3.98 eV & band gap variable, (**b**) Affinity of CdS variable & band gap = 2.4 eV.

**Figure 6:**
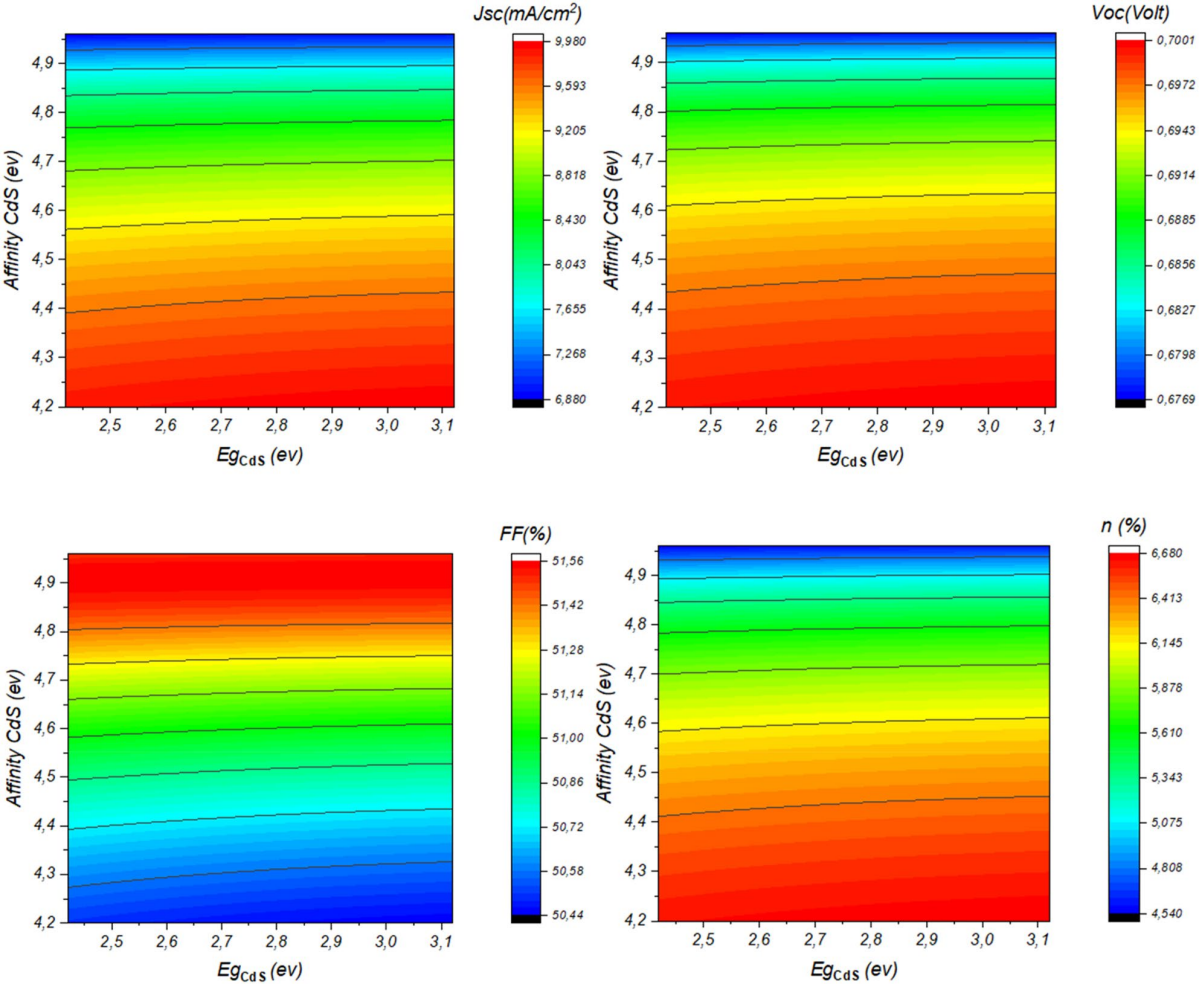
Effect of bandgap and electron affinity of CdS layer on the performance of Sb_2_S_3_ solar cell.

The bandgap and electron affinity of the CdS layer have a profound impact on Sb_2_S_3_ solar cell performance. Optimizing the bandgap enhances light absorption and energy conversion, while the electron affinity influences charge transfer and overall device efficiency. Understanding these relationships allows for the creation of more efficient Sb_2_S_3_ solar cells. Figure 5 presents the variation of device parameters as a function of CdS bandgap and electron affinity. This result is shown in detail in Fig. 6.

From Fig. 6, the significance of a high bandgap for the CdS layer becomes evident. Such a choice allows a greater number of photons to effectively penetrate the material, thereby enhancing absorption within the absorber layer. Simultaneously, a lower electron affinity plays a crucial role in improving key parameters like *J*_*sc*_ (short-circuit current) and *V*_*oc*_ (open-circuit voltage), ultimately boosting the overall conversion efficiency of the solar cell^45^. This enhancement stems from the establishment of an optimal band alignment at the CdS/Sb_2_S_3_ interface, reducing the barrier height and facilitating the smooth passage of electrons from the absorber layer to the CdS. However, it’s important to note that while both *J*_*sc*_ and *V*_*oc*_ benefit from lower electron affinity values, there is a trade-off, as the fill factor tends to decrease with decreasing electron affinity. This effect underscores the intricate balance required to optimize the performance of Sb_2_S_3_ solar cells.

### Effect of doping, thickness, bulk traps and bandgap of Sb_2_S_3_

**Figure 7:**
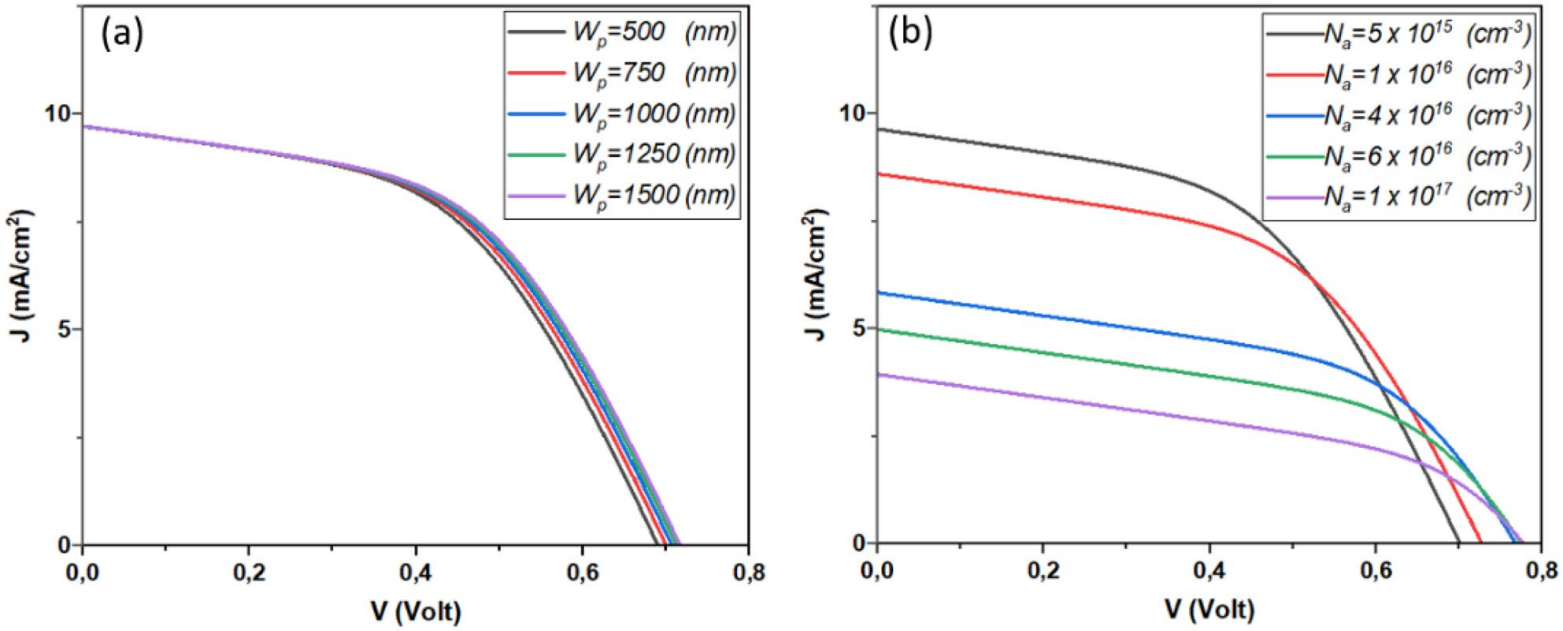
Effect of Doping, thickness of Sb_2_S_3_ layer, (**a**) *N*_*a*_ = 4.7 × 10^18^ cm^−3^ & *W*_*p*_ variable, (**b**) *W*_*p*_ = 700 nm & *N*_*a*_ variable.

**Figure 8:**
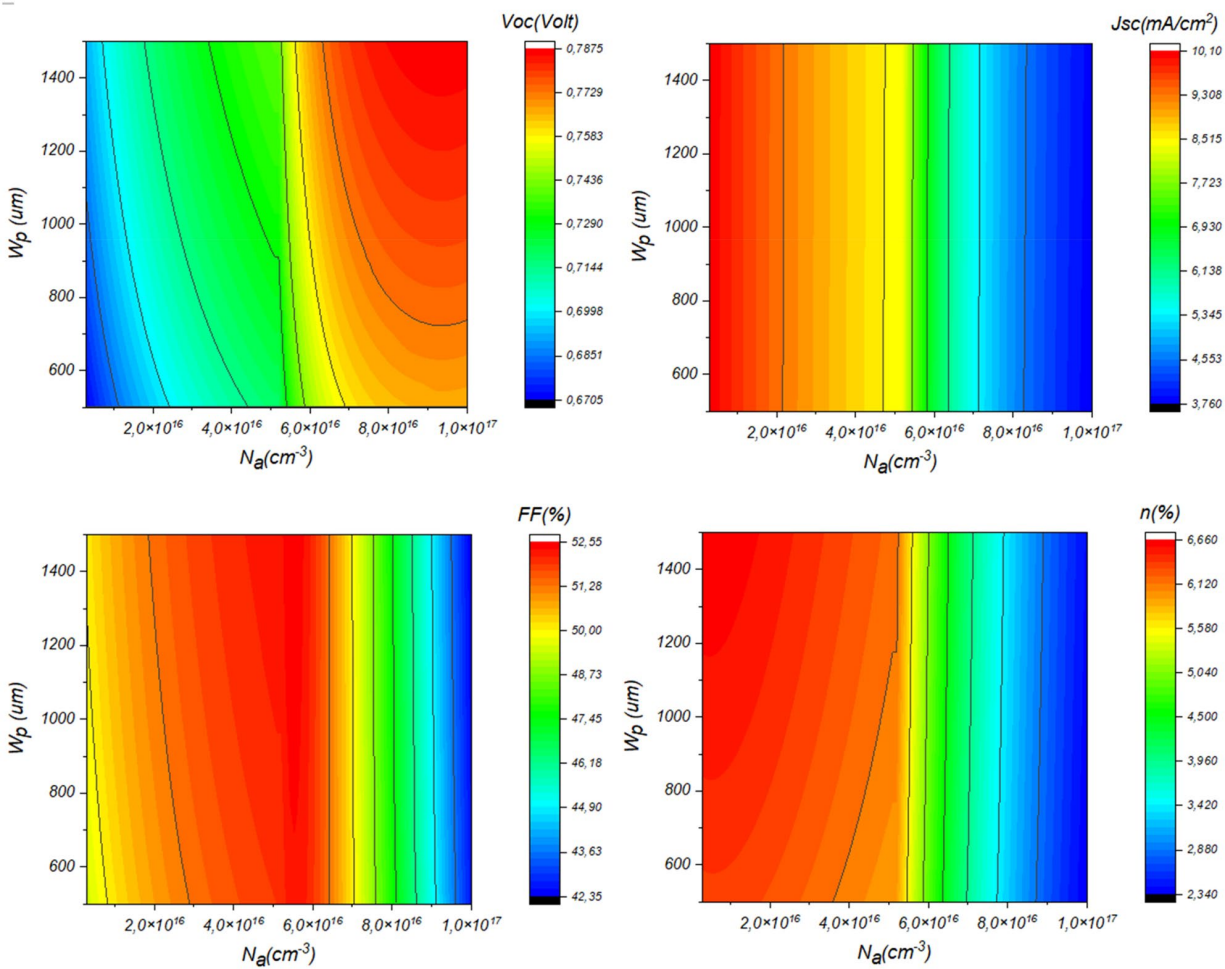
The effect of Sb_2_S_3_ layer thickness and doping concentration on solar cell performance.

Figure 7 presents the variation of the device performance as a function of Sb_2_S_3_ thickness and bandgap. This result is shown in detail in Fig. 8.

From Fig. 8, it is remarkable that for low concentration the *J*_*SC*_ will rise, where the weak concentration of the Sb_2_S_3_ layer permits the excursion of the depletion zone which enhances the electric field that leads to an efficient separation mechanism. However, low concentration leads to low *V*_*OC*_. This fact is due to the reduced reverse saturation current. Besides, the more the thickness increases the *V*_*OC*_ will enhanced owing to the enhanced photocurrent.

Another finding noted from this figure is the enhancement of the FF for a doping concentration of about 5 × 10^16^ cm^−3^. Besides, thicker Sb_2_S_3_ enhances the FF. The efficiency of the device reaches a value of 6.66% for 1 × 10^16^ cm^−3^ of concentration and 1200 nm of thickness.

**Figure 9:**
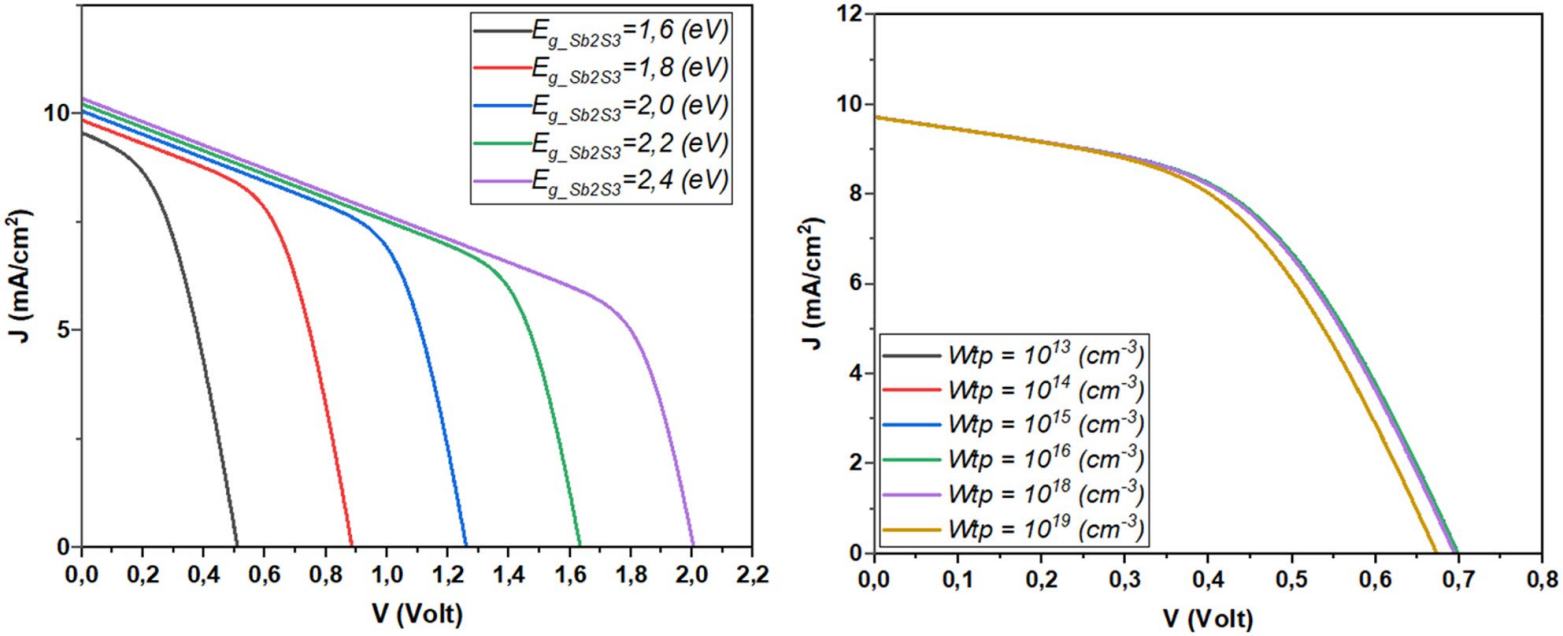
Effect of bandgap and bulk traps of Sb_2_S_3_ layer on the performance of the solar cell, (**a**) Ntp = 10^15^ cm^−3^ & *Eg* variable, (**b**) Eg = 1.7 eV & Ntp variable.

**Figure 10:**
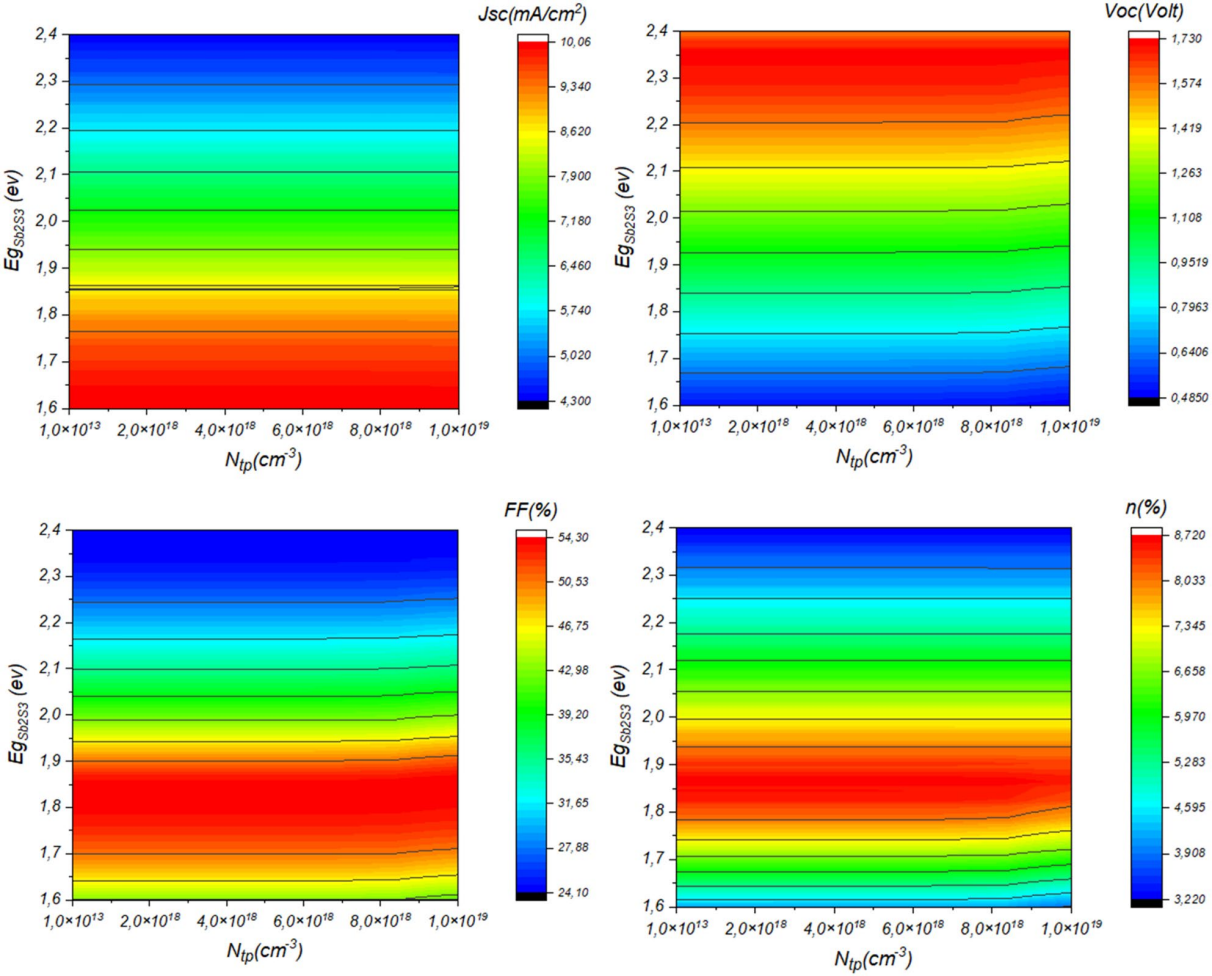
Effect of bandgap and bulk trap density of Sb_2_S_3_ layer on the performance of the solar cell.

The performance of a solar cell utilizing Sb_2_S_3_ as the absorber material is significantly influenced by two key factors: the bandgap (Eg) of the Sb_2_S_3_ layer and the density of bulk traps within the material. The bandgap determines the range of light wavelengths the material can absorb, impacting its efficiency in converting sunlight into electricity. Careful selection of the bandgap is essential to optimize absorption across the solar spectrum. On the other hand, reducing the density of bulk traps, which are defects or impurities in the Sb_2_S_3_ layer that can lead to charge carrier recombination, is crucial for improving the overall efficiency of the solar cell by minimizing recombination losses. Balancing these factors is essential in the design and fabrication of efficient Sb2S3-based solar cells. Figure 9 depicts the Effect of bandgap and bulk trap density of the Sb_2_S_3_ layer on the performance of the solar cell. This result is shown in detail in Fig. 10.

From Fig. 10, we observe that the band gap is the most influenced factor, where narrow *E*_*g*_ enhances the *J*_*SC*_ which permits the absorption of a high number of photons which enhances the generation mechanism. In contrast, the *V*_*OC*_ will be hindered due to the rise in saturation current. Another finding is the slight effect of the bulk traps on the performance of the solar cell.

### Effect of interfacial defects at CdS/Sb_2_S_3_

**Figure 11:**
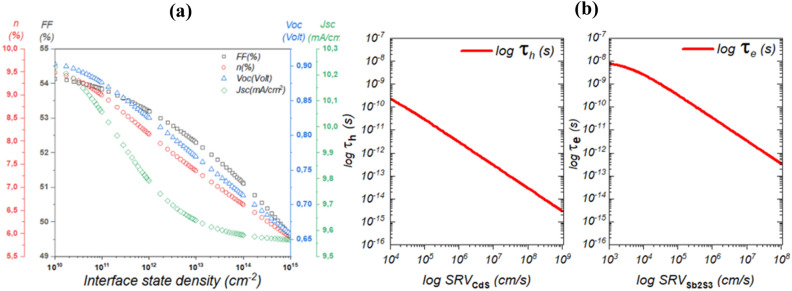
Impact of (**a**) Interface recombination density and (**b**) surface recombination velocity on the photovoltaic performance of Sb_2_S_3_ Solar Cells, deciphering the crucial factors governing device efficiency.

Interfacial defects at the CdS/Sb_2_S_3_ junction are a critical factor that profoundly influences the performance of solar cells. These defects can introduce energy level misalignments and serve as sites for charge carrier recombination, significantly limiting the efficiency of the device. Researchers delve deeply into characterizing and understanding these defects, often using advanced techniques like spectroscopy and microscopy to identify their nature and distribution. By gaining deep insights into the mechanisms underlying interfacial defects, scientists can develop strategies to mitigate their adverse effects. This includes engineering interface structures, optimizing material properties, and enhancing passivation techniques to minimize recombination and improve the reliability of the CdS/Sb_2_S_3_ interface, ultimately leading to more efficient and robust solar cell designs. Figure 11 depicts the variation of the device’s electrical outputs as a function of the interface recombination density. From this figure, it is noticeable that the interface state density has a strong influence on solar cell efficiency especially on the V_OC_, where the more the interface state density increases the more the V_OC_ decreases harshly from 0.9 V for *N*_*t*_ = 10^10^ cm^−2^ to 0.66 V for *N*_*t*_ = 10^15^ cm^−2^. This fact is due mainly favorites by the Cliff-like conformation established at the CdS/Sb_2_S_3_ interface. This conformation leads to a smaller interface bandgap and greater interface recombination.

In order to understand the effect of the interface state density, Fig. 7b,c depict the variation of minority carriers’ lifetime at both sides of the interface as a function of surface recombination velocity. It is obvious that increasing the state density will increase the SRV which hinders the minority lifetime at both sides of the interface, which leads to a harsh decrease in the performance of the solar cell. This issue can be fixed by increasing the thickness of the absorber or adopting a suitable band alignment which will certainly enhance the immunity of such device against high interface state density.

**Figure 12:**
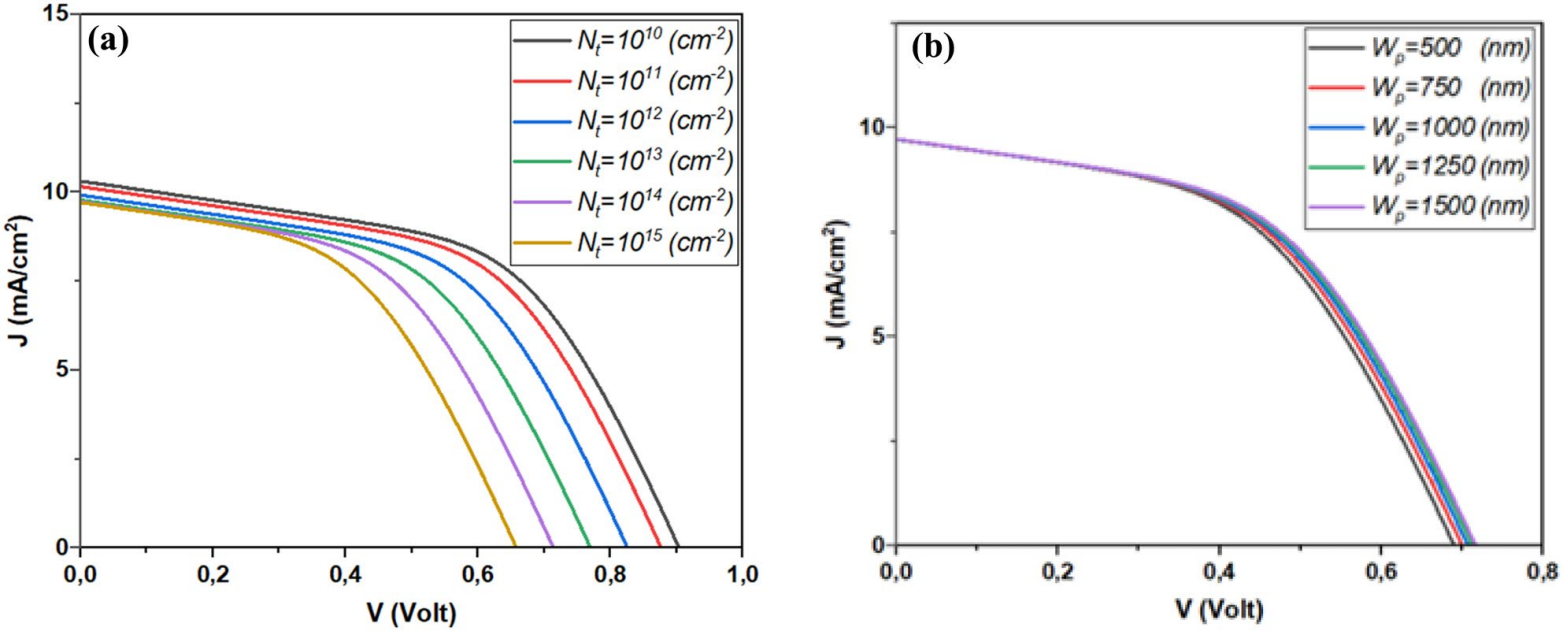
Effect of Sb_2_S_3_ thickness and CdS/Sb_2_S_3_ interface defects density on the performance of the solar cell, (**a**) *N*_*t*_ = 1.9 × 10^14^ cm^−2^ & *W*p variable, (**b**) *W*_*p*_ = 700 nm & *Nt* variable.

**Figure 13:**
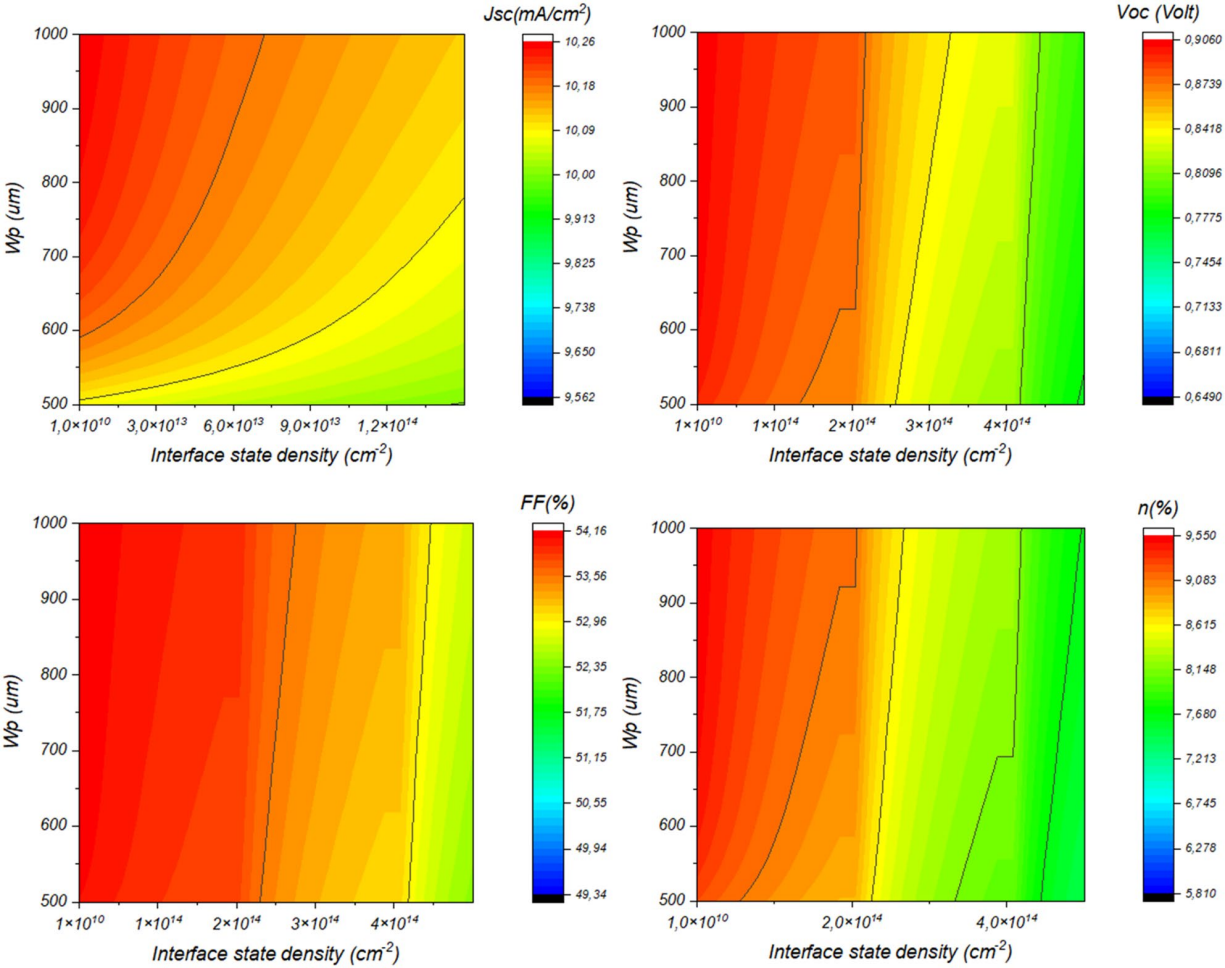
Variation of the solar cell performance as a function of both Sb_2_S_3_ thickness and CdS/Sb_2_S_3_ interface state density.

Figure 12 depicts the variation of the solar cell performance as a function of both Sb_2_S_3_ thickness and interface state density. This result is shown in detail in Fig. 13.

Figure 13 is presented here to show a strategy for overcoming the interface state density effect by compensating the trapped carriers by increasing the number of photogenerated carriers. Where, for a thickness higher than 500 nm the device can be immune to interface state density of about 2 × 10^14^ cm^−2^.

**Figure 14:**
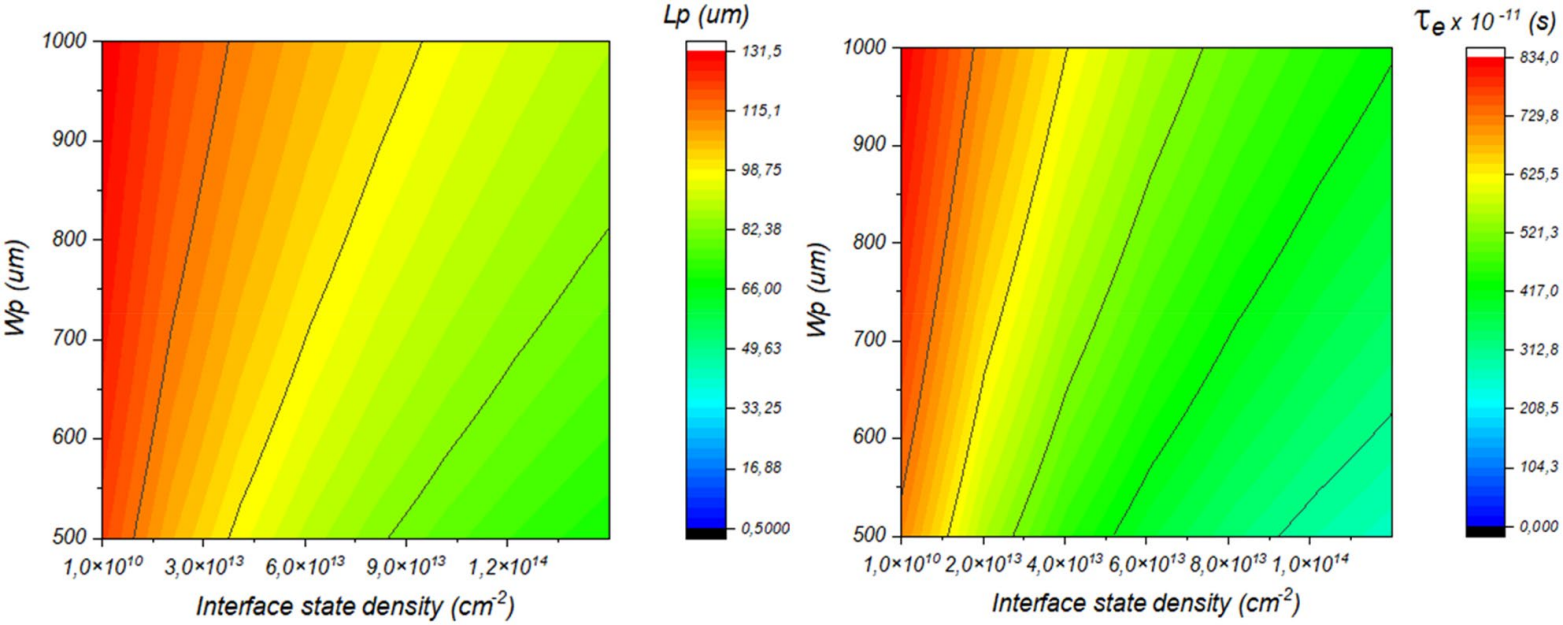
Exploring the dynamics of diffusion length and minority carrier variation in Sb_2_S_3_ Solar Cells in response to interface state density.

In order to shed light on these findings, Fig. 14 depicts the variation of both diffusion length and minority carriers as a function of interface state density. We can see from this figure the responsibility of the interface state density in reducing both diffusion length and carrier lifetime, even if we increase the thickness of the absorber the effect of such defects stays predominant. So, further enhancement by tuning both absorber thickness and interface band alignment must be performed.

### Effect of *Rsh* and *Rs*

**Figure 15:**
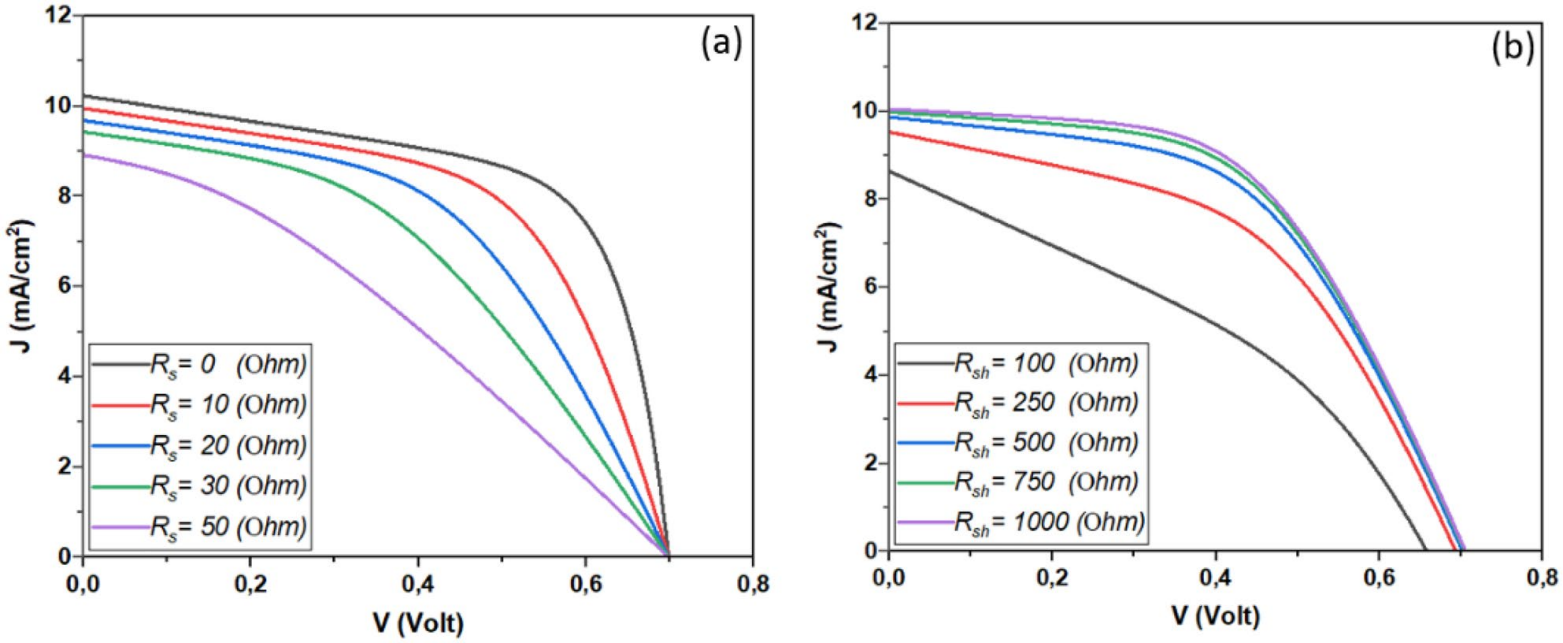
The Impact of (**a**) Series (*R*_*S*_) with *R*_*SH*_ = 351.5 Ω and (**b**) Shunt Resistances (*R*_*SH*_) with *R*_*S*_ = 18.4 Ω on Sb_2_S_3_ Solar Cell Performance.

**Figure 16:**
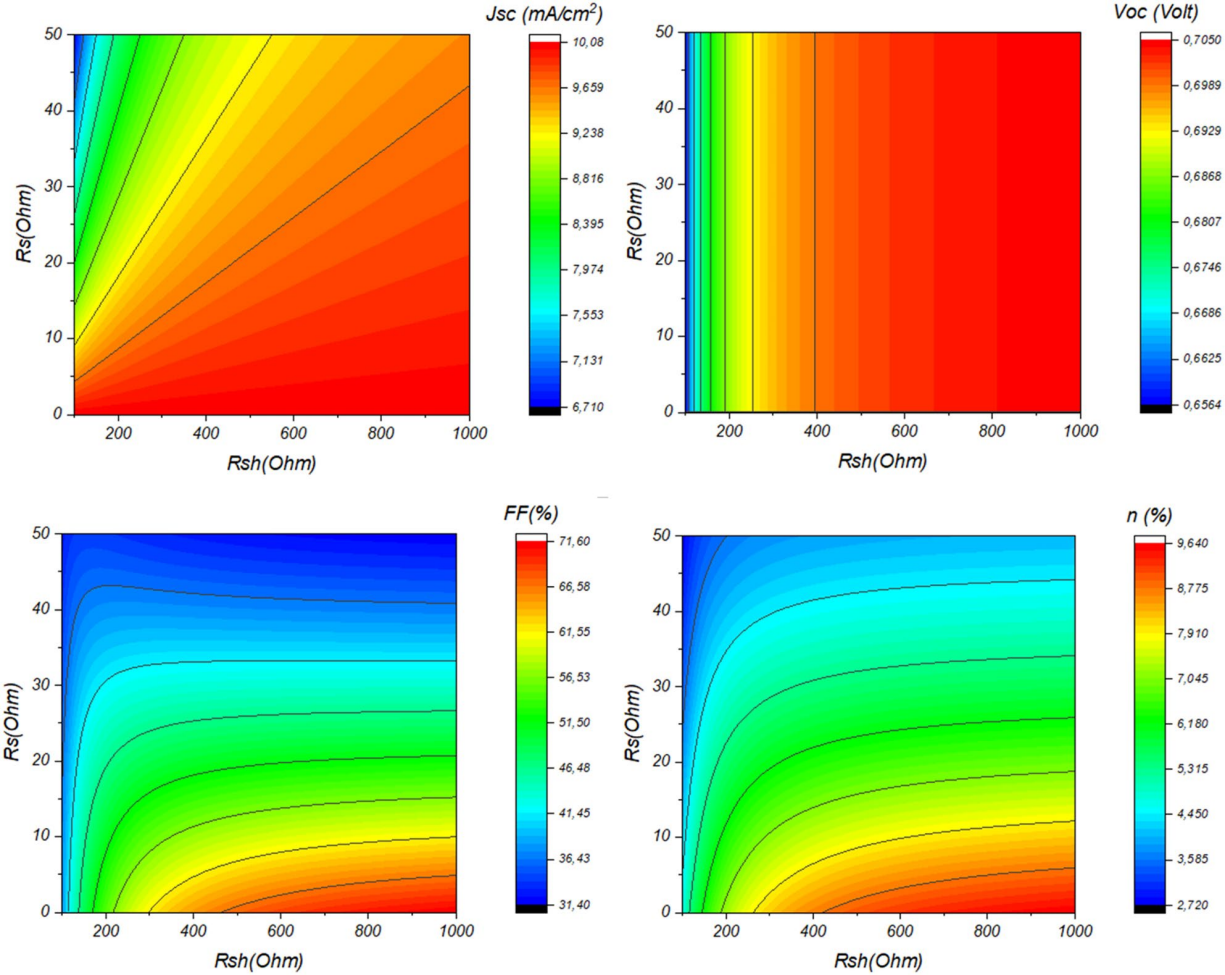
Examining the impact of series (*R*_*S*_) and shunt resistances (*R*_*SH*_) on Sb_2_S_3_ solar cell performance.

A deep understanding of the impact of shunt resistance (*Rsh*) and series resistance (*Rs*) on solar cell performance involves delving into the intricacies of electrical characteristics within the cell. Balancing *Rsh* and *Rs* is essential for optimizing solar cell performance. An ideal solar cell has high shunt resistance to minimize leakage currents while maintaining low series resistance to reduce voltage losses. Achieving this balance ensures that the cell operates at its maximum power point, optimizing both voltage and current to yield the highest power output. Figure 15 depicts the variation of the device’s electrical outputs as a function of *Rsh* and *Rs.* This result is shown in detail in Fig. 16.

The insights gleaned from Fig. 16 highlight a crucial aspect of solar cell performance: the influence of series resistance (*R*_*S*_) in conjunction with shunt resistance (*R*_*SH*_). Notably, it was observed that when R_SH_ is maintained at a high value, changes in RS have a negligible impact on both the short-circuit current (*J*_*SC*_) and the open-circuit voltage (*V*_*OC*_). However, the consequences of altering RS become pronounced when examining the fill factor (*FF*) and overall power conversion efficiency (*PCE*), which showed significant declines as R_S_ increased. This phenomenon aligns with observations in both inorganic and organic solar cells. In practical terms, R_S_ often decreases over time due to aging-related effects, leading to inconsistent photovoltaic performance. To harness the highest achievable performance, minimizing *R*_*S*_ during device fabrication becomes paramount. While one approach could involve reducing the active layer’s thickness, it’s essential to strike a balance as overly thin layers (below 200 nm) may lead to incomplete light absorption. Alternative experimental techniques to lower *R*_*S*_ include optimizing the contact resistance between the active layer and electrodes and engineering donor–acceptor interfaces that facilitate efficient charge transport^46^. These considerations underscore the intricate interplay of *R*_*S*_ and other parameters in optimizing solar cell performance.

### Optimized solar cell design

**Figure 17:**
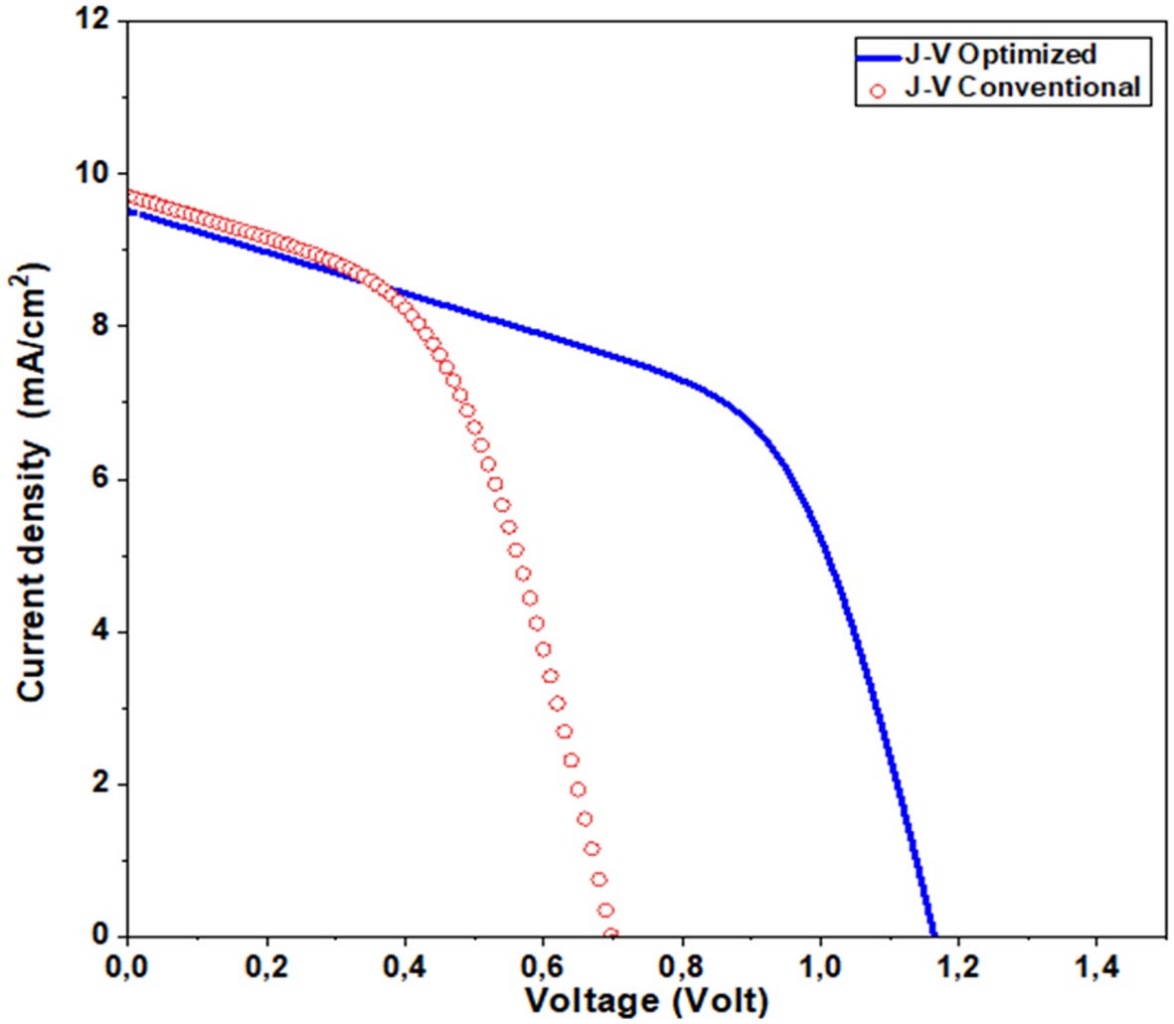
J–V characteristics of the conventional and the optimized Sb_2_S_3_ solar cell.

In Fig. 17, the I–V characteristics of both conventional^28^ and optimized solar cells are presented, revealing a substantial performance gap. Notably, the optimized solar cell surpasses the baseline in terms of an impressive open-circuit voltage (*V*_*OC*_) of 1.16 V. This remarkable enhancement can be attributed to the meticulous tuning of physical and geometrical parameters, as well as the introduction of an enhanced interface buffer/absorber layer. As a result, the optimized solar cell achieves a substantial efficiency gain, reaching an impressive 11.68% compared to the conventional solar cell’s efficiency of 6.5%. For a comprehensive overview of the results and key design parameter values, please refer to Table 4.

**Table 4:** Comparison between the optimized, experimental, and baseline solar cells.

	Structure	Exp results^28^	Baseline	Optimized
Parameters	CdS			
	Thickness (nm)	60	60	50
	Doping (cm^−3^)	–	1017	3 × 10^18^
	Bandgap (ev)	2.4	2.4	2.4
	Affinity (ev)	3.98	3.98	4.2
	Sb_2_S_3_			
	Thickness	700	700	800
	Doping	4.7 × 1015	4.7 × 1015	8 × 1015
	Bandgap	1.7	1.7	1.81
Outputs	J_SC_ (mA/cm^2^)	10.8	9.72	9.5
	V_OC_ (V)	0.71	0.70	1.16
	FF (%)	47.50	50.63	54.65
	η (%)	6.9	6.51	11.54

Table 5 demonstrates that the optimized SC outperforms state-of-the-art fabricated SCs in terms of performance metrics. Besides, by optimizing the bulk defects, interface defects, window, and ARC (Anti-Reflection Coating) materials the efficiency of our design can increase efficiently.

**Table 5:** Benchmarking table.

Device Structure	η (%)	Refs.
Sb_2_S_3_/ZnS	24.81	^47^
Sb_2_S_3_/CdS	16.53%	^48^
Sb_2_(S_1−x_Se_x_)_3_/CdS	20.15%	^49^
TiO_2_/Sb_2_S_3_	6.06%	^50^
CdS/Sb_2_S_3_	3.01%	^51^
CdS/Sb_2_S_3_	11.54	This work

## Conclusions

In conclusion, the Schockley–Quisser (SQ) limit of 28.64% remains a distant target from the current state-of-the-art power conversion efficiency (*PCE*) of 8.00% achieved in Sb_2_S_3_ solar cells. This suboptimal efficiency primarily arises from significant interface-induced recombination losses attributed to defects at the interfaces and energy level misalignments. The focus of this study was to explore an efficient Sb_2_S_3_ solar cell structure using a precise analytical model. Our proposed model, which aligns well with experimental results (Glass/ITO/CdS/Sb_2_S_3_/Au), scrutinized various parameters such as thickness, doping, electronic affinity, and bandgap. We conducted a comprehensive analysis of the impact of bulk traps in CdS and Sb_2_S_3_ on solar cell performance. Additionally, we delved deeply into the influence of interfacial traps on key solar cell parameters, including carrier minority lifetime, diffusion length, and surface recombination velocity. Our findings reveal that increasing doping and thickness enhances generation and separation mechanisms by boosting the electric field and absorption rate. However, this increment can hinder device performance through increased recombination mechanisms, including tunneling-enhanced and SRH recombination, alongside higher series resistance. Effective parameter tuning can overcome these challenges and enhance device performance. Furthermore, achieving favorable band alignment, particularly by adjusting the CdS electron affinity to establish a spike-like conformation, enhances device reliability against interfacial traps. The optimized solar cell configuration (Glass/ITO/CdS/Sb_2_S_3_/Au) demonstrates significant improvements with a high *J*_*SC*_ of 9.5 mA cm^−2^, a *V*_*OC*_ of 1.16 V, a fill factor (*FF*) of 54.7%, and a remarkable 30% increase in conversion efficiency compared to conventional solar cells. Notably, the optimized Sb_2_S_3_ solar cell not only exhibits superior performance but also demonstrates enhanced reliability in mitigating interfacial traps at the CdS/Sb_2_S_3_ interface, thanks to improved band alignment control and parameter optimization. In conclusion, the simulated work not only sheds light on the current limitations and possibilities but also lays the foundation for future research directions. The proposed scope involves a multidisciplinary approach, encompassing material science, engineering, and experimental validation, with the ultimate goal of significantly increasing the performance of Sb2S3 solar devices and bringing them closer to the Schockley–Quiser limit.

## Data Availability

The datasets used and/or analyzed during the current study are available from the corresponding author on reasonable request.
